# FPG Score: A Rapid Admission‐Based Tool for Predicting In‐Hospital Mortality in Elderly Hip Fracture Patients

**DOI:** 10.1111/os.70079

**Published:** 2025-05-15

**Authors:** Marcello Covino, Guido Bocchino, Maria Beatrice Bocchi, Chiara Barbieri, Benedetta Simeoni, Antonio Gasbarrini, Francesco Franceschi, Giulio Maccauro, Raffaele Vitiello

**Affiliations:** ^1^ Emergency Department Fondazione Policlinico Universitario Agostino Gemelli IRCCS Rome Italy; ^2^ Università Cattolica del Sacro Cuore Rome Italy; ^3^ Department of Orthopedics and Rheumatological Sciences Fondazione Policlinico Universitario Agostino Gemelli IRCCS Rome Italy; ^4^ Department of Internal Medicine and Gastroenterology Fondazione Policlinico Universitario Agostino Gemelli IRCSS Rome Italy

**Keywords:** death, hip fracture, risk score, surgery, trauma

## Abstract

**Objective:**

Hip fractures in elderly patients are a major public health concern, associated with high morbidity and mortality. Early identification of high‐risk patients is crucial to guide clinical decision‐making, optimize resource allocation, and improve outcomes. However, existing risk prediction models, such as the Nottingham Hip Fracture Score (NHFS) and the Charlson Comorbidity Index (CCI), require laboratory or postoperative data, delaying risk stratification. This study aims to develop and validate the FPG score, a novel and simplified tool for predicting intrahospital mortality in elderly patients undergoing surgery for proximal femur fractures, using only admission data available at triage.

**Materials and Methods:**

This single‐center, observational cohort study was conducted in two phases: a retrospective derivation phase (2015–2019) and a prospective validation phase (2020–2022). Patients aged ≥ 65 years with proximal femur fractures (AO 31A, 31B) undergoing surgical treatment were included. Exclusions involved pathological, periprosthetic, and femoral head fractures (31C). Data on demographics, comorbidities, vital signs, and laboratory values were collected at Emergency Unit triage. The primary outcome was intrahospital mortality. Univariate and multivariate logistic regression identified predictors, and ROC analysis assessed the FPG score's predictive performance, with AUC, sensitivity, and specificity evaluated using SPSS v25 and MedCalc v18.

**Results:**

In the retrospective phase, 1984 patients (median age: 83.5 years, 28.7% male) were analyzed, with an observed intrahospital mortality of 3.8% (77 patients). The FPG score demonstrated an AUC of 0.79, outperforming NHFS and CCI. A score > 2 was associated with a > 50% mortality risk, with 61% sensitivity and 80% specificity. In the validation cohort (752 patients, 4.8% mortality), the FPG score maintained strong predictive performance (AUC = 0.751).

**Conclusion:**

The FPG score provides a rapid, objective, and clinically applicable tool for mortality risk assessment in elderly patients with hip fractures, allowing for immediate triage‐based decision‐making. Unlike NHFS and CCI, it does not require laboratory or post‐admission data, making it particularly useful in emergency settings. Its integration into clinical practice may enhance patient management, improve resource allocation, and facilitate early intervention. While the score has been validated in a single‐center study, further multicenter validation is needed to confirm its broader applicability. Future research should explore the integration of frailty indices and laboratory markers to refine its predictive accuracy.

## Graphical abstract

Development and validation of the FPG score, a novel tool for predicting intrahospital mortality in elderly patients with hip fractures. This score provides clinicians with a reliable method for early risk stratification, facilitating targeted interventions and improving patient outcomes.

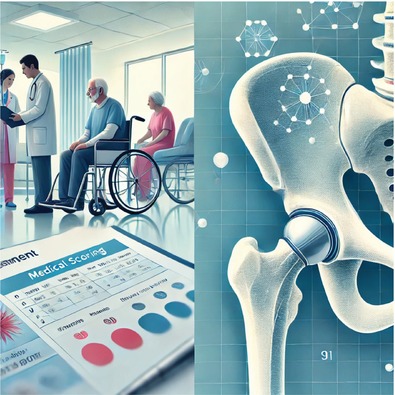

**Abbreviations**
AMA: American Medical Association
AUC: area under the curve
BP: blood pressure
BPM: beats per minute
CCI: Charlson comorbidity index
CI: confidence interval
CKD: chronic kidney disease
COPD: chronic obstructive pulmonary disease
CT: computed tomography
DM: diabetes mellitus
DVT: deep vein thrombosis
EU: emergency unit
FPG: Fondazione Policlinico Gemelli (Score)
Hb: hemoglobin
HF: heart failure
HR: heart rate
HTN: hypertension
ICU: intensive care unit
IMN: intramedullary nailing
IQR: interquartile range
IRB: Institutional Review Board
MRI: magnetic resonance imaging
NHFS: Nottingham Hip Fracture Score
NOF: neck of femur (Fracture)
OR: odds ratio
PT: prothrombin time
RCT: randomized controlled trial
ROC: receiver operating characteristic
RR: respiratory rate
SD: standard deviation
SE: standard error
SHS: sliding hip screw
THA: total hip arthroplasty

## 1 Introduction

Proximal femur fracture is a common acute medical condition whose incidence is increasing together with the aging of the world population [1, 2]. It has been estimated that the worldwide incidence of hip fractures will rise from 1.66 million in 1990 to 6.26 million by 2050, thus becoming a significant public health issue [3, 4, 5]. On average, people who experience hip fractures are 80 years old [6] and happen to be women in 80% of cases [7].

These fractures are commonly associated with a fall [8], although additional risk factors include decreased bone mineral density, degree of hip osteoarthritis, reduced level of activity, and chronic medication use [9, 10]. Hip fractures have high morbidity and mortality worldwide [11, 12, 13], extent that 12% to 17% of patients die within the first year and the long‐term risk of death is doubled in these patients [14, 15]. Outcomes following proximal femur fracture include loss of independent function and further adverse health and economic consequences for patients, their families, and finally the health care system [16, 17]. A hip fracture in an elderly patient holds indeed a severe impact on his physical and mental health, turning detrimental to his Quality of Life [18].

Most fractures are treated surgically unless the patient has significant comorbidities or reduced life expectancy [19]. The right surgery timing may affect the outcome: early surgery (within 24 to 48 h) allows earlier mobilization and rehabilitation, which speeds functional recovery and decreases the risk of medical complications [20, 21, 22]. Nevertheless, the surgical repair of hip fractures is associated with high postoperative mortality reaching up to 13.3% within the first 30 days after surgery [23].

Clinical prediction models show the reliability of factors predictors of mortality [24, 25]. To identify the high‐risk patients might be of value in aiding clinical management decisions and resource allocation [26]. The Nottingham Hip Fracture Score (NHFS) can stratify patients undergoing surgical fixation of a fractured femoral neck into high‐ and low‐risk groups, which demonstrated significant differences in mortality at 30 days and 1 year after surgery. This difference persists even when those patients who die early are excluded; however, it suggests that preoperative factors are associated with continuing mortality risk after hip fracture repair [27]. Similarly, the Charlson comorbidity index (CCI) is a system for classifying severity considering both the number and severity of 19 pre‐defined comorbid conditions, thereby generating the patient's risk of death [28, 29].

Concerning these existing clinical models, however, some limitations emerged. Many of these scores are built on data that are collected at times after the patient enters the hospital environment and often even after the surgery. Literature has also demonstrated their low specificity and sensitivity [30, 31]. This study aimed to develop a simple and effective clinical prediction model for in‐hospital mortality in elderly patients undergoing surgery for proximal femur fractures. The specific objectives were:

– To identify the key preoperative predictors of in‐hospital mortality using only admission data collected at Emergency Unit (EU) triage.
– To develop and validate the FPG score, a risk stratification tool based on easily obtainable clinical variables, ensuring high sensitivity and specificity for early mortality prediction.
– To compare the predictive accuracy of the FPG score with existing models such as the NHFS and the CCI, highlighting the limitations of current approaches and the potential advantages of the proposed model.

## 2 Materials and Methods

### 2.1 Study Population and Design

The study design included two phases: a derivation phase, involving a retrospective analysis of clinical data from patients admitted between January 1, 2015, and December 31, 2019, and a prospective validation phase conducted between January 1, 2020, and December 31, 2022.

The study was conducted to develop the FPG following the principles expressed in the Declaration of Helsinki and its later amendments. The research protocol was approved by the Institutional Review Board of Fondazione PoliclinicoUniversitario “A. Gemelli” IRCCS–Rome (#0025817/22; Study ID: #5121). A written informed consent for scientific purposes and clinical data collection was obtained according to the institutional protocol.

Clinical records of patients admitted to our EU from 1 January 2015 to 31 December 2019 with a diagnosis of proximal femur fracture deserving surgery were eligible for this study. Patients with (i) isolated greater trochanteric fractures, acetabular and pelvic fractures, (ii) pathological fractures, (iii) periprosthetic hip fractures, (iv) femoral head fractures (31C), and (v) refusal of surgical treatment were excluded from this study.

Surgical treatment was selected according to guidelines. Extracapsular proximal femur fractures (31A) were treated with either intramedullary nailing or sliding hip screws according to the fracture pattern and the surgeon's preference. Intracapsular hip fractures (31B), on the other hand, were treated with hemi or total hip arthroplasty, cannulated screws, or sliding hip screws according to the fracture pattern, the patient's seniority, and the surgeon's preference.

For the validation cohort, the consecutive patients with proximal femur fractures surgically treated were prospectively enrolled from 2020 to 2022. Exclusion and inclusion criteria were the same as in the retrospective phase.

### 2.2 Study Variables

All patients with a diagnosis of proximal femur fracture treated in the EU of our institution were managed using a standardized data collection system. Patients' characteristics were collected and registered through parameter detection at triage, blood tests at arrival, and medical history.

The variables included in the analysis were: age, sex, parameter detection at triage (heart and respiratory rate, blood pressure, and saturation), blood tests at arrival (Hb, glycemia, creatinine, creatinine clearance, nitrogen urea, fibrinogen, prothrombin time), presence on admission of cardiovascular, cerebrovascular, respiratory, or malignant diseases, and living in an institution. Pre‐existing diseases were defined based on the admission history from the patient, relatives, or notes as cardiovascular disease (pre‐existing cardiovascular conditions including previous myocardial infarction, angina, atrial fibrillation, valvular heart disease, or hypertension); cerebrovascular disease (patient has suffered a stroke or transient ischemic attack in their lifetime); respiratory disease (pre‐existing chronic respiratory conditions, including asthma or chronic obstructive airways disease but not including acute infections); renal disease (pre‐existing known renal disease, including the presence of dialysis); and malignancy (active malignancy within 20 years). Data on patients who did not undergo surgery are reported for comparison with the data used to derive the hip fracture score.

### 2.3 Measures: Outcome

The outcome modeled was the intrahospital mortality in patients with proximal femur fracture (AO 31A and 31B) who underwent surgery.

### 2.4 Statistical Analyses

#### 2.4.1 Model Evaluation Methods

Continuous variables are presented as median [interquartile range] and compared using the Mann–Whitney *U* test. Categorical variables are expressed as numbers (percentages) and compared using the Chi‐squared test, with Fisher's exact test applied as appropriate. A two‐sided *p* value of ≤ 0.05 was considered statistically significant. Study variables significantly associated with in‐hospital mortality in univariate analysis were included in a multivariate logistic regression model to identify independent predictors. Receiver operating characteristic (ROC) analysis was used to dichotomize the continuous variables before entering those in the multivariate models and to evaluate the overall discrimination value of the score. Sensitivity, specificity, and area under the ROC curve (AUC) are reported as values (95% confidence intervals). Logistic regression results are reported as odds ratio (OR) [95% confidence interval]. The goodness of fit for our model was evaluated using the Hosmer–Lemeshow test. All data were analyzed using SPSS v25 (IBM—Armonk, NY, USA) and MedCalc v18 (MedCalc Software Ltd., Ostend, Belgium).

#### 2.4.2 Score Building

Several factors were considered as predictors of intrahospital mortality and were included in the analysis in the first round. The variables considered were based on factors highlighted as significant in previously published research, and on the results of the statistical analysis.

Among the collected variables, several candidate predictors were identified by analyzing their unadjusted association with in‐hospital death, followed by multivariate analysis. Variables with fewer than five expected events were excluded from the model to avoid instability. Additionally, predictors that exceeded the threshold of five for variance inflation due to multicollinearity were condensed into a single variable. This approach was adopted to reduce the degrees of freedom in the final logistic model and to obtain more accurate estimates of the odds for each parameter. Before their inclusion in the logistic regression models, continuous variables were dichotomized using ROC analysis. To maximize predictive value, the cut‐off points for each variable were selected based on a sensitivity value of ≥ 80% for their association with the outcome.

Several vital parameters and laboratory values were missing from the dataset. Among the vital parameters, the respiratory rate had the lowest availability in the cohort. After considering the possibility of data imputation and conducting initial rounds of logistic modeling with imputed data, these variables were ultimately excluded from the final analysis to focus exclusively on clinical factors.

Similarly, laboratory data were intentionally excluded to ensure the FPG score remains a rapid and universally applicable tool that can be used immediately at triage, without waiting for test results. While laboratory markers could improve predictive accuracy, their delayed availability in emergency settings could hinder early decision making, reducing the score's practical utility.

The Fondazione Policlinico Gemelli (FPG) score was then developed based on four variables identified as independently associated with in‐hospital mortality. An automated stepwise forward multivariate logistic regression analysis was applied to the data within the creation set to construct the score. A *p* of 0.05 was set for entry into the model, and 0.1 for removal. Indicator variables were used to analyze categorical data, ensuring that all odds ratios remained greater than unity. Intrahospital mortality following hip fracture was used as the dependent variable, and the independent predictor variables were entered as covariates. Interaction between covariates was tested. The final coefficients were adjusted by rounding them to integer values to create a more easily applicable and clinically usable scoring system. Each variable was assigned a score of +1, resulting in a cumulative score ranging from 0 to 6 for each patient. The FPG score was constructed by evaluating the odds ratio of each variable to ensure a weighted yet straightforward scoring system. Based on the distribution of intra‐hospital mortality in the derivation cohort, patients were stratified into three risk categories (Table 1).

**TABLE 1:** FPGs categories.

FPGs range	Risk level	Expected mortality (%)
0–1	Low risk	1.4% (0.5%–2.3%)
2–3	Moderate risk	11.5% (6.4%–16.5%)
4–6	High risk	59.3% (36.2%–82.4%)

#### 2.4.3 Score Validation

The score performance was confirmed using a validation cohort. The validation cohort consisted of a prospective group of patients admitted with surgically treated proximal femur fractures. The overall score discrimination was evaluated by the ROC analysis. The comparison of the score's performance between the derivation and validation cohorts was assessed using the DeLong method. At the same time, the calibration of the score (i.e., the ideal direct correlation between score increases and risk of death) was assessed by comparing the score‐derived probability of poor outcome and the effective deaths in the derivation and validation cohorts. The probability of in‐hospital death based on the score was calculated by the results of a univariate logistic regression analysis of the FPG score, by the formula Expected deaths = 1/1 + *e* ^−*k*^ (with k being the coefficients derived by logistic regression analysis).

#### 2.4.4 Sample Size

As four variables were included in the logistic regression model, a total of 40 in‐hospital deaths were required in the study cohort for a satisfactory parameter estimation. Both the derivation and the validation cohort were adequate for the analysis.

## 3 Results

### 3.1 Demographics

According to inclusion and exclusion criteria, 1984 patients were considered eligible and included in the study for the derivation cohort. There were 570 (28.7%) males and 1414 (71.3%) females. The mean age was 83.5 (66–95) years old. The patients affected by extracapsular hip fracture were 1250 (63%) (M:F = 338:912), while 734 (37%) (M:F = 232:502) had an intracapsular hip fracture. The observed in‐hospital deaths were 77 in this cohort (3.8%). Baseline characteristics are shown in Table 2.

**TABLE 2:** Patient baseline characteristics.

	Total population	Alive	Death
Numbers	1984	1907 (96.1%)	77 (3.9%)
Gender (F)	1414 (71.3%)	1371 (71.9%)	43 (55.8%)
Gender (M)	570 (28.7%)	536 (28.1%)	34 (44.2%)
Mean age (years)	83.5 (66–99)	82.2 (66–95)	88.1 (74–99)
Extracapsular fractures (31A)	1250 (63.0%)	1201 (63.0%)	49 (63.6%)
Intracapsular fractures (31B)	734 (37.0%)	706 (37.0%)	28 (36.4%)
Institutionalization	53 (2.7%)	46 (2.4%)	7 (9.1%)
Hypertension	878 (44.3%)	839 (44.0%)	39 (50.6%)
Heart attack	337 (17.0%)	316 (16.6%)	21 (27.3%)
Heart failure	412 (20.8%)	379 (19.9%)	33 (42.9%)
Deep vein thrombosis	184 (9.3%)	173 (9.1%)	11 (14.3%)
Stroke	116 (5.8%)	111 (5.8%)	5 (6.5%)
Dementia	177 (8.9%)	165 (8.6%)	12 (15.6%)
COPD	111 (5.6%)	100 (5.2%)	11 (14.3%)
Diabetes	355 (17.9%)	332 (17.4%)	23 (29.9%)
Liver disease	30 (1.5%)	28 (1.5%)	2 (2.6%)
Cirrhosis	2 (0.1%)	1 (0.05%)	1 (1.3%)
Kidney failure	124 (6.2%)	103 (5.4%)	21 (27.3%)
Cancer	258 (13.0%)	250 (13.1%)	8 (10.4%)
Metastasis	31 (1.6%)	31 (1.6%)	0 (0%)
Lymphoma or Leukemia	32 (1.6%)	30 (1.6%)	2 (2.6%)
HIV	2 (0.1%)	2 (0.1%)	0 (0%)
Rheumatic disease	24 (1.2%)	24 (1.3%)	0 (0%)
Hemiplegia	9 (0.5%)	9 (0.5%)	0 (0%)
BPM		81 (60–98)	86 (61–101)
Breathing rate (per minute)		18 (12–21)	21 (13–22)
Pulse ox (SpO2%)		96 (> 94%)	95, 75 (> 94%)
Systolic blood pressure (mmHg)		144 (90–120)	138 (90–120)
Diastolic blood pressure (mmHg)		80 (60–80)	77, 17 (60–80)
Hemoglobin (g/dL)		12 (11–15.5)	11.3 (10.4–14.9)
Blood glucose (mg/dL)		123 (70–140)	134 (75–150)
Creatinine (mg/dL)		0.85 (0.5–1.7)	1.08 (0.6–2.2)
Creatinine clearance		71.369 (55.5–79.7)	52.602 (48.4–69.9)
Urea nitrogen (mg/dL)		21 (18–32)	27 (19–41)
Cr/UN		24.3 (23.2–25.1)	25.4 (23.5–26‐0)
Fibrinogen (mg/dL)		363 (298–444)	396 (276–501)
PT (seconds)		12.1 (10.9–14)	12.4 (11.1–14‐4)

Abbreviations: COPD: chronic obstructive pulmonary disease; Cr/UN: creatinine/urea nitrogen; F: female; M: male; PT: prothrombin time.

### 3.2 Comparison Between Survivors and Deceased Patients

The comparison between the two patient groups—those who survived and those who died—reveals several important differences in demographics, medical history, and clinical characteristics, which may help explain the factors contributing to mortality following hip fractures.

Age appears to be a significant factor, with the deceased group having a higher mean age (88.1 years) compared to the survivors (82.2 years). This suggests that advanced age is associated with increased mortality risk in patients with hip fractures, possibly due to frailty and a higher likelihood of comorbidities that complicate recovery.

In terms of gender, although females comprised the majority of both the total population (71.3%) and the surviving group (71.9%), a higher proportion of deceased patients were male (44.2% of the deceased compared to 28.7% of the overall population). This discrepancy could point to different mortality risks between genders, potentially influenced by other underlying health conditions or differences in how males and females respond to treatment.

Looking at the type of hip fracture, the distribution of extracapsular (63%) and intracapsular (37%) fractures was similar across both groups. Both groups had a high incidence of extracapsular fractures, with no significant difference between survivors and deceased. This suggests that fracture type alone may not be a determining factor for mortality in this study. The status of institutionalization also stood out as a notable difference, with 9.1% of the deceased patients being institutionalized compared to just 2.4% of those who survived. This suggests that individuals with a higher level of pre‐existing dependence or severe comorbidities may be at a greater risk of dying following a hip fracture, possibly due to factors like reduced mobility or impaired recovery potential.

### 3.3 Medical History and Comorbidities

When examining medical history, several chronic conditions were more prevalent in the deceased group. For instance, heart attack and heart failure were more common among those who died. Specifically, 27.3% of deceased patients had a history of heart attack, compared to 16.6% in survivors, while 42.9% of deceased patients had heart failure, compared to 19.9% in the surviving group. These cardiovascular conditions are known to increase the risk of complications, such as poor wound healing, increased infection risk, or even cardiac events during recovery, which likely contributed to the higher mortality rate.

Similarly, diabetes was more prevalent among those who died (29.9% compared to 17.4% in survivors), which could explain a higher risk of complications, including infections or delayed healing. Kidney failure was another key factor, with 27.3% of deceased patients having kidney failure compared to only 5.4% of survivors. Renal dysfunction can complicate the management of hip fractures, as it can affect the body's ability to process medications and recover from surgery, thus leading to higher mortality.

Finally, respiratory and cognitive conditions also appeared to contribute to mortality. The deceased group had higher rates of COPD (14.3% vs. 5.2%) and dementia (15.6% vs. 8.6%). These conditions often lead to poorer recovery outcomes, as patients may have compromised pulmonary function and cognitive impairments that hinder their ability to participate in rehabilitation or adhere to medical advice.

Regarding clinical parameters at EU, some differences were observed in the initial vital signs and blood tests. The deceased group had a slightly higher breathing rate (21 per minute compared to 18 in survivors), suggesting more respiratory distress upon arrival. Systolic blood pressure was marginally lower in the deceased group (138 mmHg vs. 144 mmHg), and hemoglobin levels were also slightly lower in the deceased (11.3 g/dL vs. 12 g/dL in survivors), which could indicate worse overall health or blood loss. Additionally, creatinine levels were elevated in the deceased group (1.08 mg/dL vs. 0.85 mg/dL), suggesting poorer kidney function at the time of admission, which likely contributed to the higher mortality.

### 3.4 Univariate and Multivariate Analysis

A univariate study was performed of all the variables at our disposal taken individually. The variables that emerged with statistical significance were age, institutionalization, heart failure, diabetes, and kidney failure. These variables were then combined for a multivariate logistic regression analysis that resulted in the exclusion of heart failure.

The variables analyzed as predictors of intra‐hospital mortality on univariate analysis are shown in Table 3. Based on the premises described in the methods section, the FPG score value was calculated for each patient.

**TABLE 3:** Multivariate logistic regression.

	*p*	Odds ratio	CI 95% inferior	CI 95% superior
Age	0.000	1.120	1.084	1.158
Institutionalization	0.001	4.013	1.817	8.861
Diabetes	0.001	2.264	1.384	3.703
Kidney failure	0.000	3.421	1.914	6.114
Heart failure	0.729	0.909	0.530	1.560

### 3.5 Development of the FPG Score

The FPGs assess the risk of intrahospital mortality following hip fracture surgery in patients aged > 65 years. The risk model is based on four variables: age, institutionalization, diabetes, and kidney failure. Between 0 and a maximum of 2 points is scored for each variable, resulting in a sum score of the FPGs ranging from 0 to 6 points. Going into more detail of our model is shown in Table 4.

**TABLE 4:** FPGs criteria and points.

Category	Criteria	Points
Age	< 65 years old	0
	65–84 years old	1
	≥ 85 years old	2
Institutionalization	No institutionalization	0
	Living in an institution (e.g., care home or nursing home)	1
Diabetes	No diagnosis of diabetes on arrival	0
	Diagnosis of diabetes on arrival	1
Kidney failure	No diagnosis of kidney failure on arrival	0
	Kidney failure without dialysis	1
	Kidney failure with dialysis	2

### 3.6 Area Under the ROC Curve

The area under the ROC curves (SD) of the NHFS, CCI, and the FPGs is respectively 0.73 (standard error 0.03; *p* = 0.000), 0.73 (standard error 0.03; *p* = 0.000), and 0.79 (standard error 0.03; *p* = 0.000) (Table 5).

**TABLE 5:** Area under the ROC curve.

Scoring system	AUC	Standard error	95% Confidence interval	*p*
NHFS	0.73	0.03	0.713–0.752	< 0.001
CCI	0.73	0.03	0.712–0.752	< 0.001
FPGs	0.79	0.03	0.769–0.805	< 0.001

### 3.7 Youden Index

Youden's index was used to optimize the use of FPGs. For a value greater than 2, the score demonstrated a sensitivity of 61% and a specificity of 80%.

### 3.8 Prospective Validation Phase

In the second phase of the study, we analyzed a population comparable to the derivation cohort, aiming to validate the FPG score. This validation phase included a prospective cohort of 752 patients admitted between 2020 and 2022 with surgically treated proximal femur fractures. Inclusion and exclusion criteria were identical to those applied during the derivation phase, ensuring methodological consistency.

The population had a mean age of 84 years (range 67–99), with females constituting 71.5% (538/752) and males 28.5% (214/752). Among the patients, 716 (95.2%) survived, and 36 (4.8%) died, mirroring the proportions observed in the derivation cohort. Institutionalization was reported in 3.2% (24/752), while 9.7% (73/752) had dementia, 4.8% (36/752) had COPD, and 8.4% (63/752) were diabetic. The prevalence of kidney failure was similarly low, at 2.3% (17/752).

These demographic and clinical characteristics demonstrate the methodological consistency between the derivation and validation cohorts, reinforcing the comparability of outcomes and the robustness of the FPG score as a predictive tool.

### 3.9 Score System Development

The data were observed by assigning the FPG scores to all analyzed patients, representing the observed values. Using the methods described in the previous sections, the model was constructed to calculate the expected results. Table 6 highlighted the relationship between FPG scores and patient survival, revealing a clear trend: as the FPG score increased, both observed and predicted mortality rates rose significantly.

**TABLE 6:** Contingency table of FPGs in the validation cohort and predictive mortality rates by FPGs.

FPG score value	Numbers	Observed alive and %	Observed deaths and %	Deaths predicted by the FPGs
0	249	247 (99.2%)	2 (0.8%)	0.8%
1	323	315 (97.5%)	8 (2.5%)	2.3%
2	126	113 (89.8%)	13 (10.2%)	6.4%
3	38	32 (84.2%)	6 (15.8%)	16.5%
4	12	8 (66.7%)	4 (33.3%)	36.2%
5	3	1 (33.3%)	2 (66.7%)	62.0%
6	1	0 (0%)	1 (100%)	82.4%
Total	752	716	36	

For patients with an FPG score of 0, the mortality rate was remarkably low, at just 0.8%, indicating an excellent prognosis. This trend remained favorable for patients with scores of 1 or 2, where mortality rates were relatively low at 2.5% and 10.2%, respectively. However, as the FPG score increased to 3 and beyond, the observed mortality rose sharply. For instance, an FPG score of 3 corresponded to a 15.8% mortality rate, while a score of 4 was associated with a one‐in‐three chance of death (33.3%).

This progression continued with scores of 5 and 6, where mortality rates reached 66.7% and 100%, respectively. A key strength of this scoring system lay in its predictive power, as demonstrated by the close alignment between the observed mortality rates and the predicted values. The model was particularly effective in identifying high‐risk patients, as scores of 4 and above corresponded to a mortality risk exceeding 30%, with scores of 5 and 6 signaling critical levels of risk.

## 4 Discussion

This study developed and validated the FPG score, a simplified tool for predicting in‐hospital mortality in elderly patients undergoing surgery for proximal femur fractures. Based on four preoperative variables (age, institutionalization, diabetes, and kidney failure), it showed strong predictive performance (AUC 0.79 in derivation, 0.751 in validation). A score > 2 indicated a > 50% mortality risk (61% sensitivity, 80% specificity), outperforming NHFS and CCI. Unlike these models, the FPG score enables immediate triage‐based risk stratification, facilitating early intervention and optimized clinical decisions.

### 4.1 Mortality Risk in Hip Fracture Patients

Mortality in the elderly patient population increases 4% per year [32]. Going specifically to femur fractures, the first year appears to be the most critical with a 5‐fold and 8‐fold increase respectively for women and men within the first 3 months as compared with age‐ and sex‐matched controls [32, 33]. Because of these poor outcomes, proximal femoral fractures in frail older patients can be seen as a life‐threatening condition. Approximately 1.3 million persons worldwide fracture the proximal femur annually. Due to the progressive aging, this number is expected to increase worldwide to the extent that the annual costs will reach about $131 billion [34]. In 2017, The Dutch National Institute for Public Health and the Environment reported a total healthcare cost for hip fractures of € 130 M for men and € 331 M for women (0.48% of the total yearly healthcare expenditure), an increase of 17% from 2007 [35, 36, 37]. All of this will imply significant changes in the quality, quantity, and structure of health care services putting higher pressure on health care budgets.

Preoperative prediction of mortality in hip fracture may guide which patient may benefit from surgery. Identifying patient characteristics associated with mortality may aid surgeons, patients, and families in shared decision‐making and optimize care in hip fracture patients with significant health and socioeconomic implications [38, 39].

### 4.2 Limitations of Existing Risk Prediction Models

Medical predictor models have been developed over the past few years. The ideal risk‐scoring tool should meet the following prerequisites: simple, easy to use, reproducible, accurate, reliable, objective, and available to all patients. However, a rather recent qualitative systematic review on this matter pointed out that the predictive accuracy of the medical predictors available could be more robust and that multinational validation is currently lacking [31].

For these reasons, the purpose was to develop a clinical prediction model for intrahospital mortality in elderly patients with proximal femur fractures surgically treated.

The NHFS and the CCI use readily available pre‐operative data. The CCI is a medical risk prediction tool, which has been adapted for surgical risk stratification. It uses well‐defined comorbidities; however, it is based solely on anamnestic clinical data, thus on exclusively subjective variables.

The NHFS is a combination of seven independent objective predictors of mortality, but not without limitations. Hemoglobin values, for example, are highly dependent on the hydration status of the patient, and it is well known that elderly patients tend to drink less for multiple reasons even without considering the “hospitalization factor.” Still, the positive history for active malignancies without further distinction does not consider the possibility of tumors, such as hematologic tumors to name a few, for which there is a pharmacological therapeutic option and thus an all‐around favorable prognosis. The NHFS is furthermore a hip fracture‐specific score.

### 4.3 Clinical Implications of the FPG Score

The FPGs consisted of the following 4 independent predictor variables: age, institutionalization, diabetes, and kidney failure.

Older patients are particularly vulnerable, as age is closely associated with increased mortality risk, both during hospitalization and in the post‐fracture period. Previous studies confirm that age is the primary determinant of mortality in elderly patients with traumatic hip fractures [40]. Mortality rates are notably higher in patients over 85 years compared to those aged 75–85 years [41]. Furthermore, age strongly influences the likelihood of institutionalization: each additional decade of age increases the risk of institutionalization approximately 2.5 times [42]. Physical recovery post‐fracture also plays a crucial role, with patients exhibiting poor post‐fracture physical function showing a fivefold increased risk of institutionalization compared to those with higher physical performance levels [42].

Diabetes represents another critical factor in mortality risk following femoral neck fractures. Diabetic patients experience significantly poorer outcomes compared to their non‐diabetic counterparts. One‐year survival rates are 68.0% for diabetic patients versus 87.3% for non‐diabetic patients (*p* = 0.033). Among diabetic patients, predictors of mortality include advanced age, postoperative complications, and elevated HbA1c levels, underscoring the combined impact of diabetes‐related metabolic imbalances and the challenges of surgical recovery in this population [43].

Chronic kidney disease (CKD), particularly in its advanced stages, is a third major determinant of mortality in patients with femoral neck fractures. Bone metabolism alterations, such as cortical bone loss caused by hyperparathyroidism and elevated turnover, are common in CKD long before renal replacement therapy begins [44]. As a result, patients with CKD face an increased fracture risk [45], with those in stage 4 CKD showing significantly higher mortality following hip fractures [45]. Among dialysis patients, the one‐year post‐fracture mortality rate is 55%–64%, a 2.7‐fold increase compared to non‐fractured dialysis patients [46, 47].

This model makes it possible to calculate, for this selected category of frail patients, the risk of in‐hospital mortality in an extremely rapid and statistically reliable manner.

A rapid history collection at triage on arrival in the emergency department is sufficient, in most cases, to establish the risk of intrahospital mortality for patients with proximal femur fracture with indication for surgical treatment. FPGs are based solely on anamnestic data that can be quickly collected at triage, making it a simple and rapid tool to categorize immediate mortality risk in this population. This allows clinicians to quickly initiate critical interventions after a hip fracture, optimizing patient outcomes. The timing of surgery plays a crucial role in these outcomes, as early surgery promotes quicker mobilization and rehabilitation, which in turn speeds up functional recovery and minimizes the risk of medical complications [20, 21, 22].

### 4.4 Comparison With Existing Models

**FIGURE 1:**
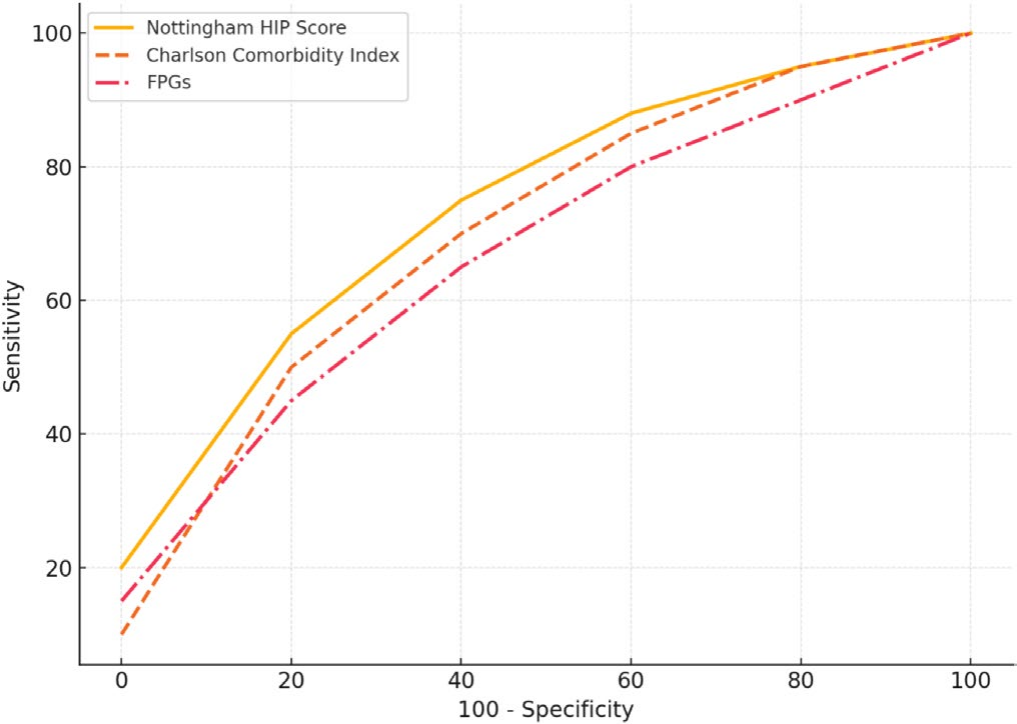
ROC curve comparison.

The NHFS and the CCI have both shown reasonable, though not excellent, discriminant characteristics for mortality and morbidity and have been validated external to their original cohort (Figure 1).

Until that point, the NHFS had demonstrated the best results in terms of discrimination and calibration for predicting early mortality following hip fracture surgery. However, it exhibited limited discriminative power and inconsistent calibration results.

Based on the area under the curve, the FPG risk model showed excellent discriminative value. An FPG score greater than 2 predicted the probability of in‐hospital mortality in elderly patients with hip fractures exceeding 30%, with a sensitivity of 61% and a specificity of 80%.

### 4.5 Limitations and Strength

This study has several limitations that should be acknowledged. First, the FPG score was developed and validated within a single‐center setting, which may limit its generalizability to different healthcare systems and patient populations. Although the prospective validation confirmed the model's robustness, further multicenter studies are necessary to ensure its applicability across diverse clinical environments. Second, the model does not incorporate a frailty index, which is increasingly recognized as a critical factor in postoperative outcomes. Given the high prevalence of frailty among elderly hip fracture patients, integrating frailty assessment tools into the FPG score could further refine its predictive accuracy. Additionally, the score does not include biochemical markers, such as inflammatory or nutritional parameters, which may provide additional prognostic value. Future research should explore whether incorporating such variables enhances the model's predictive performance without compromising its simplicity and immediate applicability in emergency settings. Despite these limitations, this study presents several strengths that highlight the clinical utility of the FPG score. Unlike existing models such as the NHFS and CCI, the FPG score relies solely on four easily obtainable preoperative variables (age, institutionalization, diabetes, and kidney failure). This makes it a simple, objective, and immediately applicable tool that can be used at triage without requiring laboratory results or postoperative data. Additionally, the prospective validation demonstrated consistent predictive accuracy (AUC 0.751), reinforcing the model's reliability. Another key strength of this study is the high specificity (80%) of the FPG score, which reduces the likelihood of false‐positive risk stratification, allowing for more precise resource allocation and early clinical intervention. Finally, given its ease of implementation, the FPG score has the potential to be widely adopted in emergency settings, improving decision‐making efficiency and ultimately enhancing patient outcomes. Future research should focus on large‐scale external validation in multicenter cohorts, as well as the integration of frailty indices and biochemical markers to further optimize the score's predictive capabilities.

## 5 Conclusion

The aim of the study was to develop an easy‐to‐use clinical prediction model capable of determining the mortality of patients with surgically treated proximal femur fracture as early as through data obtainable on admission at EU triage reliable in sensitivity and specificity. This model has shown an excellent discriminative value and by a score > 2 is able to predict the probability of intrahospital mortality in elderly patients with hip fracture > 50% with a sensitivity of 61% and a specificity of 80%. The FPG score represents a valuable tool for rapid risk stratification in elderly patients with hip fractures. Its integration into clinical practice may enhance patient management, improve resource allocation, and ultimately lead to better patient outcomes. Further validation on a multinational scale is essential to confirm its reliability and broader applicability.

## Author Contributions

**Marcello Covino:** conceptualization, data curation, investigation, writing – original draft, writing – review and editing. **Guido Bocchino:** conceptualization, data curation, writing – original draft, writing – review and editing, methodology, software. **Maria Beatrice Bocchi:** software, data curation, validation, formal analysis, visualization, writing – original draft, writing – review and editing. **Chiara Barbieri:** project administration, resources, visualization, writing – original draft, writing – review and editing, formal analysis, validation. **Benedetta Simeoni:** methodology, investigation, validation, formal analysis, funding acquisition, writing – original draft, writing – review and editing. **Antonio Gasbarrini:** conceptualization, validation, formal analysis, supervision, resources, writing – original draft. **Francesco Franceschi:** software, methodology, data curation, validation, funding acquisition, writing – original draft, writing – review and editing. **Giulio Maccauro:** conceptualization, validation, supervision, project administration, writing – original draft, resources, writing – review and editing. **Raffaele Vitiello:** conceptualization, data curation, investigation, validation, supervision, visualization, project administration, resources, writing – original draft, funding acquisition.

## Disclosure

The authors have nothing to report.

## Conflicts of Interest

The authors declare no conflicts of interest.
